# Cryogenic Wavelength-Multiplexed Eight-Channel Optical-to-Microwave Converter

**DOI:** 10.1021/acsphotonics.6c00869

**Published:** 2026-07-15

**Authors:** Dominik Bisang, Stefan M. Koepfli, Daniel Rieben, Lewin Bormann, David Moor, Shadi Nashashibi, Laurenz Kulmer, Michael Baumann, Marina Homs, Yuriy Fedoryshyn, Juerg Leuthold

**Affiliations:** 31060 ETH Zurich, Institute of Electromagnetic Fields, Zurich 8092, Switzerland

**Keywords:** cryogenic, photonic link, wavelength demultiplexing, silicon photonics, plasmonic, graphene photodetector

## Abstract

Next-generation superconducting quantum computers face scalability limitations due to the increasing number of required interconnects from room temperature into the cryogenic environment. Scalability can be improved by transitioning to photonic links, and even further scaling can be obtained by employing wavelength multiplexing. This would allow to replace multiple RF cables by a single optical fiber. However, experimental demonstration of a cryogenic multichannel optical-to-microwave converter, required for demultiplexing incoming optical signals and performing conversion to microwave signals inside the cryostat, is still missing. Here, we demonstrate the first cryogenic eight-channel optical-to-microwave converter. It combines an arrayed waveguide grating in silicon photonics for wavelength demultiplexing with eight waveguide-integrated plasmonic graphene photodetectors. The device has a compact footprint of 1.75 mm × 0.71 mm, enabling scalable and dense integration. We characterized all eight channels at 4 K and found electrical crosstalk below 30 dB, high bandwidth >45 GHz, subnanoampere dark current, and 4–5× increased responsivity compared to room temperature. Finally, as a proof of concept, we simultaneously transferred two RF signals modulated on two different optical carriers into the cryostat and achieved crosstalk isolation of >50 dB in the best case. Therefore, our device demonstrates the potential of wavelength-multiplexed photonic links for mitigating the cryogenic interconnect bottleneck.

## Graphical abstract

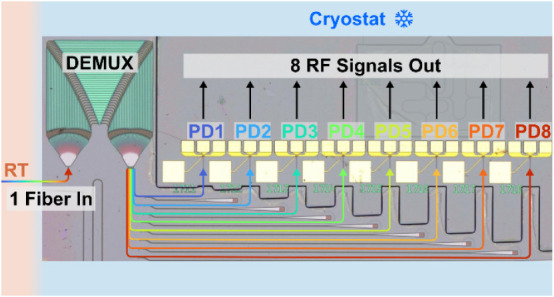

## Introduction

Photonic links into cryogenic environments are essential for future quantum computing systems.^1–3^ With the number of qubits in superconducting quantum computing circuits growing, the number of interconnects between room temperature (RT) and cryogenic environments needs to increase as well. Currently, coaxial radio frequency (RF) cabling is used for signal transfer. While this works well for tens to hundreds of qubits, larger systems with tens of thousands of qubits will face limitations^1–4^ due to heat transferred by the cabling, noise introduced by the required attenuators, size of the RF connectors, and cost of the cabling and filtering components. Therefore, optical fibers have been proposed^1–3^ since they offer low thermal conductivity, low noise, low footprint, and low cost. To send control signals from RT into the cryostat, an RF signal would be created at RT, modulated onto an optical carrier, and transported into the cryostat by an optical fiber. By having a photodetector (PD) after the fiber, the received optical signal can be converted back to an electrical signal and applied to the quantum circuit. Recent demonstrations using photonic links with cryogenic PDs have shown successful control and readout of superconducting qubits.^1,5^

Cryogenic PDs for photonic links from RT to a cryogenic environment should at least fulfill the following requirements: The PD needs to be compatible with cryogenic temperatures, which is not necessarily given due to temperature-dependent material and interface parameters. Next, the PD needs sufficient bandwidth for the application, since the signals for superconducting qubits are typically in the region of 1–10 GHz,^6^ with proposals for extending to higher frequencies for relaxed temperature requirements.^7^ Further, the responsivity should be sufficiently high to reduce the required optical input power, and the dark current should be low to decrease Joule heating. Both will reduce the power consumption within the cryostat, where only limited cooling power is available. Several PDs operating at cryogenic temperatures have been demonstrated so far based on InGaAs,^1,8–14^ graphene,^15^ germanium,^16^ and silicon.^14^ High bandwidth of >110 GHz has been shown by a graphene-based PD,^15^ where simultaneously an increase in responsivity by 4.2× compared to RT was reported. A high responsivity of 850 mA/W at 1550 nm was demonstrated by a 10 GHz extended-InGaAs PD.^13^ Dark currents below 10 pA have been shown by several demonstrations.^1,13–16^ A table listing the cryogenic high-speed PDs found in literature, together with the reported key metrics, is given in Supporting Information Note 1.

While impressive single PDs and single-channel photonic links have been demonstrated so far, scaling to thousands of photonic links will require further measures. Among other approaches, several researchers have suggested putting the PDs to a 4 K stage and connecting to a mK stage by superconductive cables,^2,3^ rather than having the PDs at a mK stage.^1^ This is mainly due to the active heat load of the PDs during operation, which limits the number of PDs at the mK stage. Putting the PDs to a 4 K stages could still enable more connections than would be possible with only conventional RF cabling.^2,3^ In terms of PD size, demonstrated photonic links so far used bulky off-the-shelf components,^1,13,14^ but in the future, densely integrated devices will be required. Silicon photonics can provide excellent scalability^17,18^ since it allows for integrated photonic devices with a low footprint, and the large-scale manufacturing capabilities of CMOS can be employed. Further, to enable the transport of even more RF signals into the cryostat and push scaling limits further, optical wavelength multiplexing has been proposed.^1–3,13,19^ Since an optical fiber offers a usable bandwidth of several THz, many RF signals can be multiplexed onto optical carriers at different wavelengths. By having a wavelength demultiplexer inside the cryostat, the signals can be split up to different photodetectors, where the individual RF signals will be recovered. Such an envisioned system is conceptually visualized in Figure 1(a).

**Figure 1:**
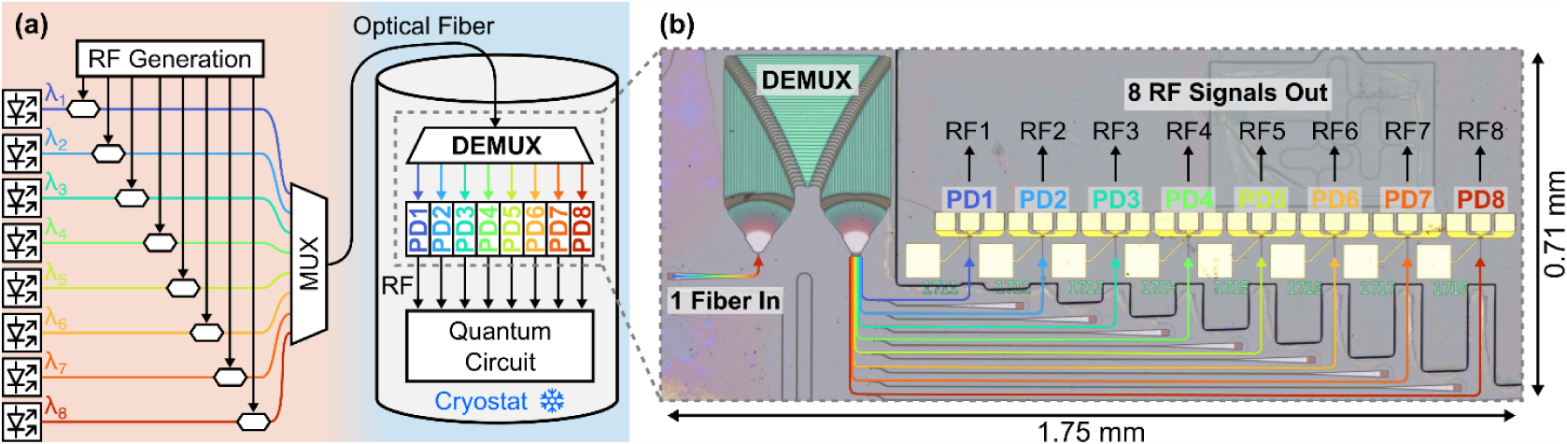
(a) Envisioned photonic wavelength-multiplexed link into a cryostat. (b) Microscope image of the implemented integrated device consisting of an optical wavelength-demultiplexer (DEMUX) with eight waveguide-integrated PDs.

Cryogenic optical wavelength (de)-multiplexing must exhibit low insertion loss, as optical losses lead to unwanted heating. In addition, crosstalk between the channels should be minimized to prevent signal impairments. From a system-level perspective, optical multiplexing in a photonic link has the advantage, that 1 dB optical crosstalk isolation will result in 2 dB electrical crosstalk isolation. This is due to the subsequent PD, where 1 dB difference in optical power entering a PD corresponds to 2 dB difference in electrical power generated in the PD. A demonstration with electrical frequency-multiplexing of control signals for superconducting qubits has achieved channel isolation of 30–40 dB,^20^ yet higher isolation can be expected by employing optical multiplexing. Lastly, the device footprint should be small for dense integration. In silicon photonics for room-temperature applications, wavelength (de)-multiplexing is often implemented with ring filters, but since they require active tuning with thermal heaters having power consumption in the order of tens of mW,^21,22^ they are unsuitable for scalable cryogenic operation. Instead, fully passive structures such as arrayed waveguide gratings (AWG), echelle gratings, or integrated Bragg reflectors can be used.^23^ Since these multiplexing structures require optical waveguides, the PDs need to be waveguide-integrated as well.

However, integrated wavelength (de)-multiplexing at cryogenic temperatures is barely experimentally explored.^19,24^ Also, among the high-speed PDs characterized at cryogenic temperatures, only a few are waveguide-integrated,^12,16^ as would be required for a scalable approach combined with on-chip wavelength demultiplexing. As such, while photonic links with cryogenic wavelength-multiplexed photodetectors have been proposed as a solution for scaling to thousands of interconnects, experimental demonstration of even one cryogenic multichannel optical-to-microwave converter is still missing.

In this paper, we demonstrate an eight-channel optical-to-microwave converter operating at 4 K, consisting of a 1 × 8 wavelength-demultiplexer with waveguide-integrated PDs at each of the 8 demultiplexer outputs. The demultiplexer was implemented as an AWG in silicon photonics, and microwave-to-optical conversion was achieved using waveguide-integrated plasmonic graphene PD. A microscope picture of our device with a compact footprint of only 1.75 mm × 0.71 mm is shown in Figure 1(b). We characterized the device at 4 K, and found all channels working with low crosstalk, subnanoampere dark current in each PD, and >45 GHz bandwidth in each channel. Finally, we simultaneously transferred two RF tones modulated on two neighboring optical channels into the cryostat using a single fiber, and showed low crosstalk between the received signals with the best achieved isolation of more than 50 dB.

## Results and Discussions

### Optical Wavelength Demultiplexing

We implemented the optical wavelength demultiplexing using an AWG in silicon photonics. AWGs have the advantage that they can be operated fully passively, i.e., do not require active tuning by thermal heaters. Further, they are well established at RT in literature.^23,25–27^ A microscope picture of such a 1 × 8 AWG can be seen in Figure 2(a). The device has a compact footprint of 410 μm × 450 μm and is fabricated on a silicon-on-insulator (SOI) substrate. More fabrication details are provided in Supporting Information Note 2.

**Figure 2:**
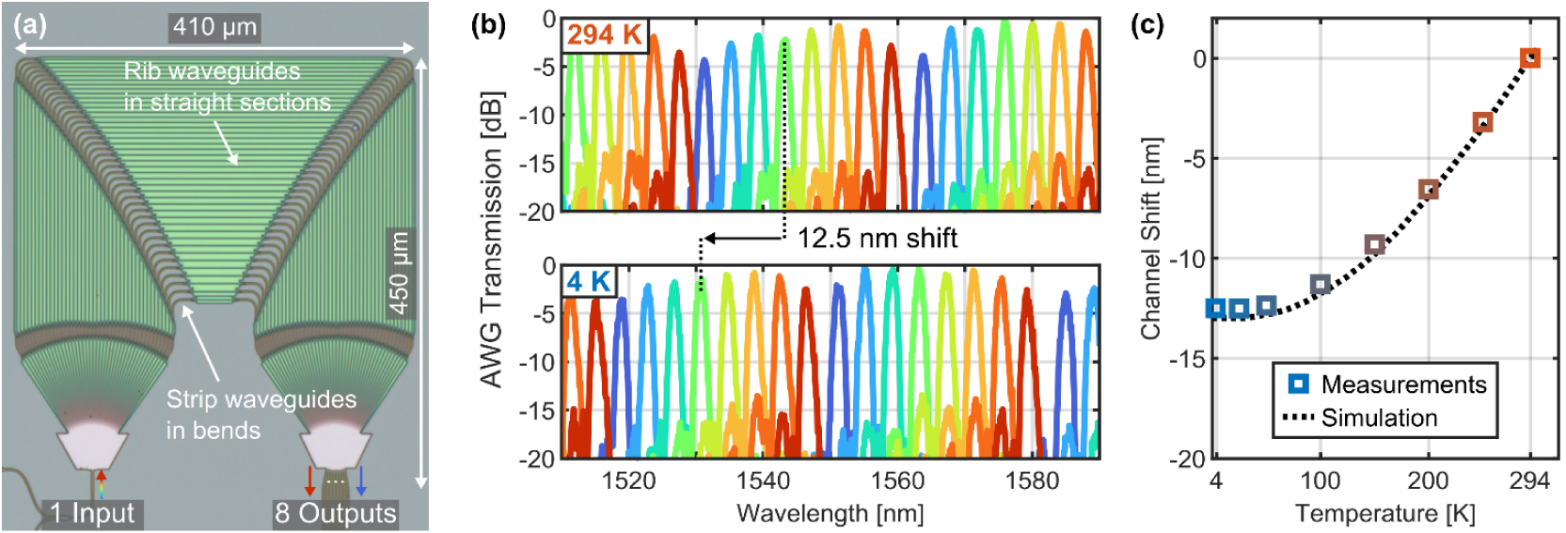
(a) Microscope image of a 1 × 8 AWG for wavelength demultiplexing with compact footprint. (b) Transmission spectrum of the AWG at temperatures of 294 K (top) and 4 K (bottom). A shift of about 12.5 nm in the transmission spectrum can be observed when cooling down the AWG from 294 to 4 K. (c) Simulated and measured channel shift of the AWG depending on temperature.

For the design of the AWG, we used rib waveguides with a 700 nm wide rib in the straight sections of the AWG arms, since they are more fabrication tolerant than strip waveguides. This yields a more consistent effective mode index and therefore less unwanted phase fluctuations between the arms of the AWG, resulting in reduced crosstalk. In the bends of the arms, we used strip waveguides, since these allow for a smaller bend radius, resulting in reduced device footprint. Conversion between rib and strip waveguides was achieved by a 7.5 μm long taper. We designed the AWG to have a cyclic transmission spectrum, where the free spectral range (FSR) equals the channel spacing multiplied by the number of channels. This compensates for an expected shift of the transmission spectrum during cooldown from RT to 4 K, where by having a cyclic AWG, spectral gaps are avoided and channels will be available at any temperature in the wavelength area of interest.

We characterized the AWG shown in Figure 2(a) by measuring the optical transmission from the input to each of the 8 outputs. Figure 2b shows the measured AWG transmission of each channel over wavelength at RT of 294 K (top), and at a cryogenic temperature of 4 K (bottom). Each color corresponds to one of the eight outputs. Grating coupler losses from fiber-to-chip coupling have been removed by measuring the coupling losses using a reference device. At 294 K, we found an AWG insertion loss of 1–4 dB depending on the channel, a 4 nm channel spacing and 32 nm FSR as per design, a 3 dB-bandwidth around 1.4 nm (approximately 87 GHz for the electrical signal), and optical crosstalk around 15 dB. These values are comparable with other silicon photonics AWGs found in literature.^23,26,27^ When comparing the measurements at 294 K with those at 4 K, we found that most metrics do not change significantly with temperature, except for an expected shift in channel wavelengths by 12.5 nm as marked by dotted lines.

To investigate the AWG channel shift over temperature, we have measured channel number 4 (light green curve in Figure 2(b)) at various temperatures and plotted the shift of the channel wavelength over the temperature range in Figure 2(c). From RT down to 150 K, we observed a linear shift of the channel wavelength. Below 150 K, the channel shift started to slow down, with nearly no shift below 50 K. This behavior is mainly attributed to the temperature-induced change in the refractive index of silicon, which shows the same qualitative trend.^28^ Using temperature-dependent refractive index data from literature on silicon^28^ and silicon dioxide,^29^ we have modeled the channel shift over temperature (Supporting Information Note 3). We found good agreement between simulated and measured data, as shown in Figure 2(c). This indicates that AWGs are indeed well understood in the literature, and design equations can be translated to cryogenic temperatures. These are important insights for future device engineering of cryogenic wavelength demultiplexers.

### Plasmonic Graphene Photodetector

We used waveguide-integrated plasmonic graphene PDs in this work for the detection of optical signals and the subsequent conversion to RF signals in the cryogenic environment. Graphene PDs have several advantages, making them beneficial for this work: They work at cryogenic temperatures with even improved performance compared to RT,^15^ can have high bandwidths exceeding 100 GHz^30–34^ due to graphene’s inherent carrier dynamics,^35–37^ can be used without a bias voltage applied^38^ which results in a low dark current only from gate leakage, and can be postprocessed on top of silicon photonics in a back-end-of-the-line process^39–44^ for compatibility with the silicon photonics wavelength demultiplexer.

We first review the working principle of our PD.^45,46^ A false-colored SEM image of our PD is shown in Figure 3(a), and a schematic cross-section through the PD is shown in Figure 3(b). Light is guided from the AWG to the PD in a silicon photonics waveguide and then absorbed in a bilayer sheet of CVD-grown graphene. Since graphene is atomically thin, it has only a small volume and the optical absorption is usually very low, resulting in a low responsivity. To improve the responsivity, we use plasmonic resonators made of gold, which create a local field enhancement leading to higher absorption in the graphene. A simulation of the optical E-field magnitude in the photodetector with clearly visible field enhancement is shown in Figure 3(c). For the extraction of the photogenerated carriers from graphene, electrical contacts to the graphene are needed. The gold resonators serve as signal electrode, and additional silver electrodes are used as ground electrodes. These two different metals contacting the graphene create a bending of the energy band of graphene,^33^ resulting in carrier extraction even when no additional bias voltage is applied (*V*_Bias_ = 0 V). Finally, the Dirac point position of the graphene strongly depends on the deposition, defects, contamination, and substrate; and can vary from device to device. To compensate for this, a top gate made of gold was deposited over the active area of the PD, with a dielectric alumina spacer in between. For each PD and temperature, the gate voltage *V*_Gate_ was swept between 20 V and +25 V, and the gate voltage yielding the largest photocurrent was subsequently used.

**Figure 3:**
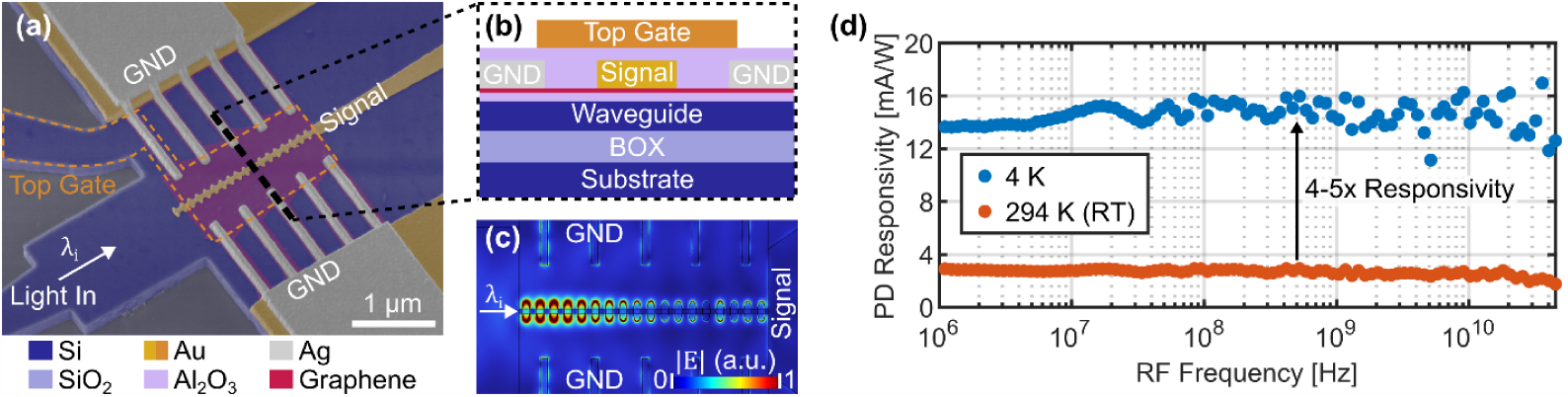
(a) False-colored SEM image of a waveguide-integrated plasmonic graphene PD. (b) Schematic cross-section of the PD. (c) Simulated optical E-field in the PD with plasmonic enhancement at the resonators. Light is entering the PD from the left side. (d) Responsivity vs RF frequency of one PD at RT and 4 K. When cooling down from RT to 4 K, the responsivity increases by 4–5×.

The measured responsivity of one PD at 294 and 4 K is shown in Figure 3(d). The responsivity is presented as a function of RF frequency between 1 MHz and 45 GHz, when only a gate voltage *V*_Gate_ and no bias voltage is applied (*V*_Bias_ = 0 V). As can be seen, the responsivity increased by a factor of 4–5× when cooling the PD down from RT to 4 K. This is in line with a previous characterization of a cryogenic graphene PD, where also the origin of this increase was investigated.^15^ Further, the measurement at 4 K shows a near-flat frequency response over the measurement range. The data shown in Figure 3(d) is from the PD in channel 8 of our device, showing average responsivity. Here, the influence of the AWG and fiber-to-chip coupling has been removed to get the responsivity of only the PD. For these measurements, the setup shown in Figure 4(a) and described later in the text was used. Here, we want to highlight that the setup enables the application of a bias voltage *V*_Bias_ between signal and ground, which has been kept to *V*_Bias_ = 0 V since the bias current would result in significant dark current due to the low-resistive graphene channel. In fact, our measurements have shown that at cryogenic temperatures, *V*_Bias_ = 0 V was the near-optimal biasing condition for the highest photocurrent. This is in stark contrast to room-temperature operation, where usually a bias voltage is required for maximum photocurrent. The resulting dark current of less than 200 pA was therefore only arising from gate leakage.

**Figure 4:**
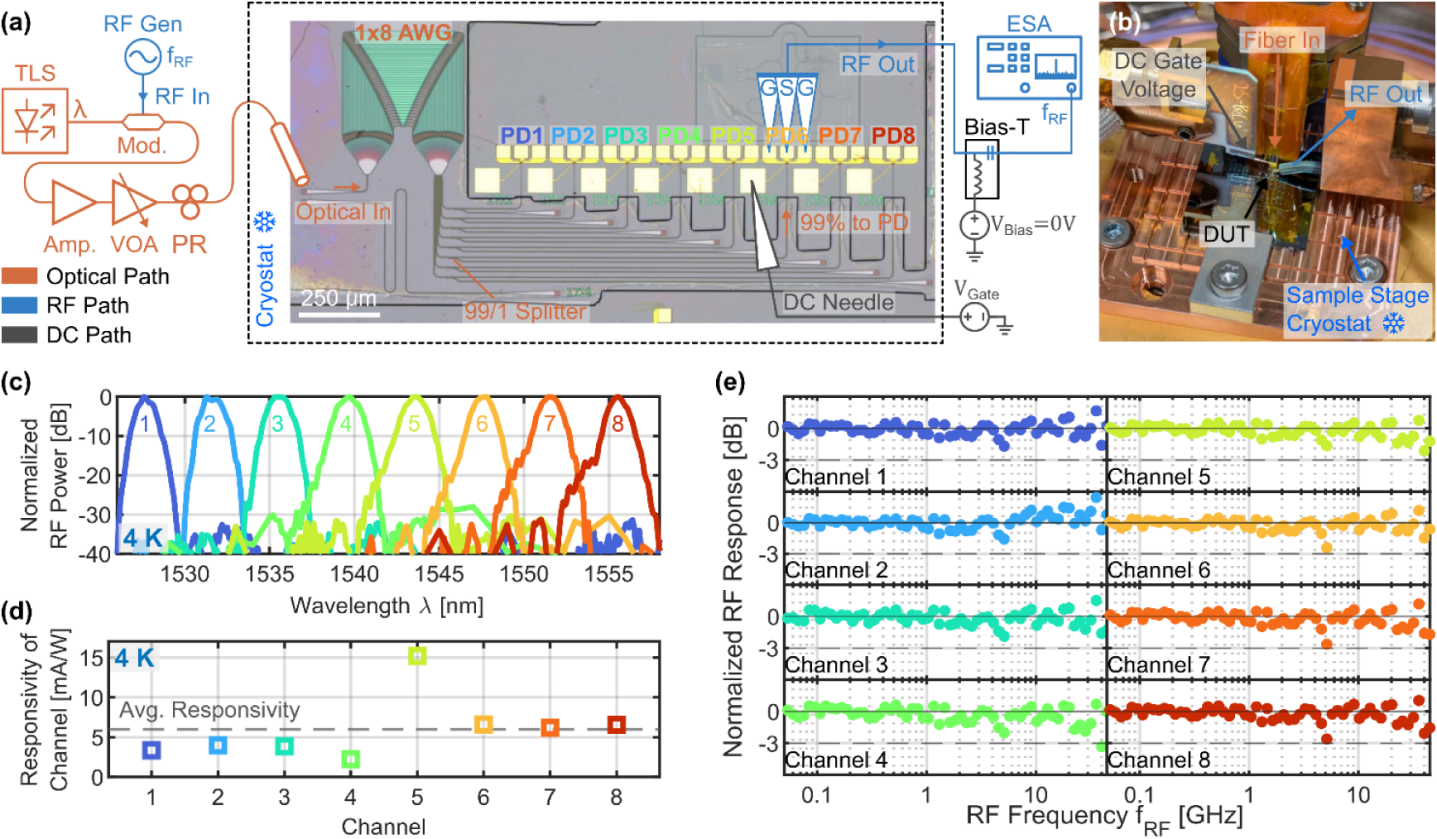
(a) Microscope picture of the DUT, a cryogenic eight-channel wavelength-multiplexed optical-to-microwave converter, together with a setup schematic for characterization of the DUT. (b) Photograph of the DUT in contact with probes and fiber array for measurements inside the cryostat. (c) Measured spectral response of each channel at 4 K, where the normalized RF power of a *f*_RF_ = 5 GHz tone generated in the PD is shown as a function of the optical wavelength. (d) Extracted responsivity of each channel at 4 K at the corresponding peak wavelength. The on-chip channel responsivity captures the AWG insertion loss and the PD responsivity, while the fiber-to-chip coupling loss is subtracted. The dashed line shows the average responsivity over all eight channels. (e) Normalized RF response as a function of applied RF frequency for all channels at 4 K. All channels show a near-flat response between 50 MHz and 45 GHz.

### Cryogenic Eight-Channel Optical-to-Microwave Converter

In this section, we present our cryogenic wavelength-multiplexed eight-channel optical-to-microwave converter, enabled by cointegrating eight plasmonic graphene photodetectors with a silicon photonics 1 × 8 AWG for wavelength demultiplexing.

We first describe the measurement setup and the device under test (DUT). In Figure 4(a), a microscope picture of the DUT is shown, together with a schematic of the setup used for characterization of the device. The fabrication of the device is discussed in Supporting Information Note 2. For these measurements, we used a tunable laser source (TLS) that emits a single optical carrier with wavelength λ. A single-tone RF frequency *f*_RF_ was then modulated onto the optical carrier using an external modulator with a bandwidth of 30 GHz. The optical signal was then amplified, attenuated in a variable optical attenuator (VOA) for controlling the exact power level, and then going through a polarization rotator (PR) optimized for maximum fiber-to-chip coupling. After the PR, the signal was fed into the cryostat by a fiber feedthrough, and then coupled to the chip by a grating coupler inside the cryostat. A cryo-compatible piezo was used for fine-positioning of the fiber. The on-chip power after the grating coupler was 5–8 dBm, depending on the wavelength. After coupling, the optical signal was guided by silicon waveguides to the 1 × 8 AWG for wavelength demultiplexing. Compared to the AWG shown in Figure 2(a), we have slightly optimized the design of the AWG used here, resulting in about 5 dB lower optical crosstalk. At each output of the AWG, there is a 99/1 splitter where 1% of the signal was coupled out of the chip for direct characterization of the AWG. The other 99% was guided to a waveguide-integrated plasmonic graphene PD, where the optical signal was converted to an RF signal inside the cryostat. We contacted the PDs with a GSG RF probe and brought the signal out of the cryostat through an electrical RF feedthrough. Outside of the cryostat, a bias-T was connected, which would have allowed for the application of an additional bias voltage *V*_Bias_, but this was kept to *V*_Bias_ = 0 V as discussed in the previous section and was only used to monitor the DC photocurrent. The RF signal at frequency *f*_RF_ was then recorded in an electrical spectrum analyzer (ESA). Additionally, for optimal operation of the graphene PD, we applied a gate voltage *V*_Gate_ via a DC needle. Figure 4(b) shows a photograph, where the on-chip DUT was contacted by the fiber array, RF probe and DC gate needle. The chip was clamped to a copper sample holder, which was mounted on the sample stage of the 4 K cryostat.

We then characterized each channel of the DUT individually. First, we measured the spectral response of each channel by sweeping the TLS wavelength λ and recording the generated RF signal. The result is shown in Figure 4(c). Clearly, the signals have low electrical crosstalk, with isolation between the channels of 30 dB and more at the central wavelengths. This is in line with the optical crosstalk measurement of the AWG shown in Figure 2(b), since 1 dB difference in optical power entering a PD corresponds to 2 dB difference in electrical power generated in the PD, as explained earlier. Next, we show the measured responsivity of each channel in Figure 4(d). The channel responsivity consists of the channel-dependent AWG insertion loss and the responsivity of each PD. It was measured by comparing the recorded signal against a measurement with a known reference PD and subtracting the fiber-to-chip coupling loss. The average channel responsivity is 6 mA/W, indicated by the dashed line in Figure 4(d). Six of the eight channels have similar performance, with two outliers in channels 4 and 5. We attribute the low responsivity of the graphene PDs as the main reason for the low channel responsivities. The PD responsivity can be further increased by using an improved PD design, as we have demonstrated with a waveguide-integrated graphene PD achieving 68 mA/W at RT in the C-band.^45^ We discuss the impact of the measured 6 mA/W channel responsivity on the active heat load of our device in Supporting Information Note 4, and show that the active heat load can be massively reduced. Regarding the channel-to-channel variations, we note that these are undesired, since they complicate system-level control by introducing different losses to each channel. Still, as of now, superconducting qubits anyways require extensive calibration per channel.^47^ We therefore assume that the channel-to-channel variations of our device pose no bottleneck for scalability. The variations arise most likely from local differences in the graphene introduced during transfer and processing of the graphene, and can be reduced by the continuous improvements in scalability and consistency in graphene-based fabrication.^42,44,48^ Finally, we show the measured frequency response of each channel in Figure 4(e). For these measurements, we have set the wavelength λ to the peak wavelength of each channel and swept the RF modulation frequency *f*_RF_ from 50 MHz to 45 GHz. Clearly, all channels show a near-flat frequency response over this range.

To conclude the DUT characterization, we have shown eight channels working at cryogenic temperatures with low crosstalk between the channels, subnanoampere dark current in each PD, and high bandwidth in each channel.

### Multiplexed Two-Tone Transfer into Cryostat

To demonstrate the viability of our device, we simultaneously transferred two RF tones modulated onto two different optical carriers into the cryostat with a single fiber, and showed high isolation between the channels. This experiment mimicked how two channels of our device would be used in a real-world use-case.

We first describe the setup used for this experiment. Figure 5(a) shows a simplified schematic of the used setup, which is essentially a subset of the envisioned setup depicted in Figure 1(a). A more detailed setup schematic is shown in Supporting Information Note 5. First, we selected channels 6 and 7 of our device for the demonstration. We have selected these two channels,

**Figure 5:**
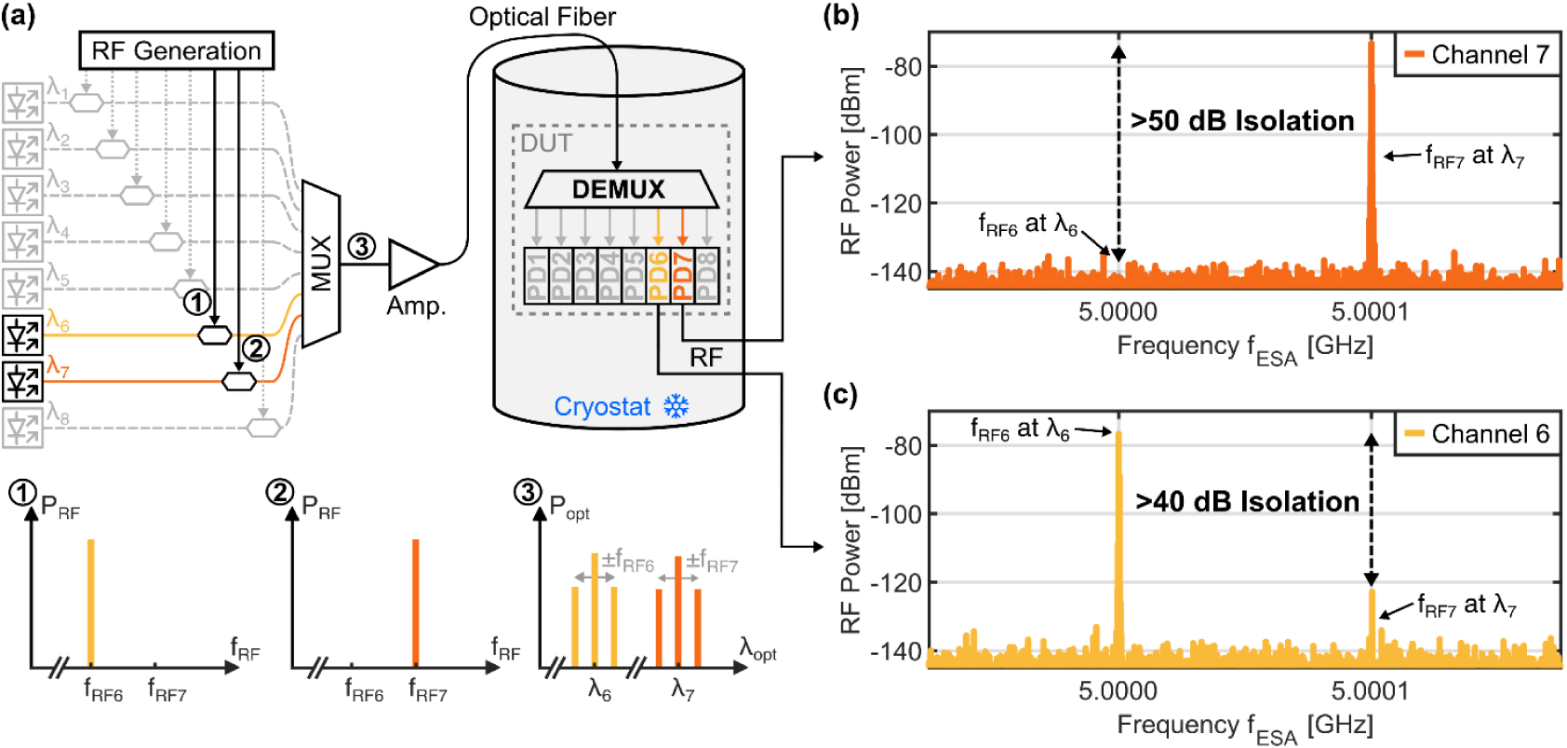
(a) Simplified schematic of the setup used for simultaneously transferring two RF tones encoded onto two different optical carriers into the cryogenic environment by a single fiber. The insets ① and ② conceptually show the generated RF tones and inset ③ conceptually shows the optical signal spectrum after modulation and wavelength multiplexing. (b,c) Measured received electrical power spectrum in (b) PD7 and (c) PD6. High isolation >40 dB between the channels is achieved.

since they have shown similar performance in terms of responsivity (Figure 4d) and are neighboring channels which typically would show worst crosstalk. We then generated two RF tones *f*_RF6_ = 5 GHz and *f*_RF7_ = 5 GHz +100 kHz = 5.0001 GHz at RT (conceptually depicted in insets ① and ② in Figure 5(a)). In a real application using superconductive qubits after the PDs, the two qubits might, but not necessarily need to, use the same frequencies. In our experiment, we chose a typical qubit frequency of 5 GHz for both tones, but in order to distinguish the two received signals in the ESA, we used a 100 kHz offset for *f*_RF7_. These signals are then modulated onto two optical carriers at wavelength λ_6_ = 1551.5 nm and λ_7_ =1555.5 nm, which are the peak wavelengths of channel 6 and 7, respectively. Afterward, they were combined using an optical 50:50 coupler. The optical spectrum of the resulting signal is conceptually depicted in inset ③ in Figure 5(a). The multiplexed optical signal was then amplified and fed into the cryostat. There, it was coupled into the DUT, where the signal got demultiplexed in the AWG and received in the PDs. We measured the electrical spectrum generated in each PD using an ESA.

We now present the measurement results. Figure 5(b) shows the measured spectrum of PD7, where clearly the target tone at *f*_RF7_ = 5.0001 GHz was retrieved. The tone at *f*_RF6_, which was modulated onto λ_6_ and intended for another channel, was vanishing in the noise floor, thereby isolated by more than 50 dB. Figure 5(c) shows the same situation for PD6. Here, the tone at *f*_RF6_ was clearly visible as intended, and *f*_RF7_ was isolated by more than 40 dB. The measured channel isolation from this experiment is in agreement with the crosstalk measurement presented in Figure 4(c). Overall, our device was capable of demultiplexing the incoming optical signal with isolation of >50 dB in the best case, highlighting the potential of this approach.

## Conclusion

We presented the first wavelength-multiplexed eight-channel optical-to-microwave converter working at cryogenic temperatures. First, we showed a silicon photonics 1 × 8 AWG for wavelength demultiplexing which provides compact footprint of 410 × 450 μm, insertion loss of 1–4 dB, and optical crosstalk below 15 dB. During cooldown from 294 to 4 K, no significant change in performance occurred except a shift of the channel peak wavelengths by 12.5 nm, which can be accurately modeled. Then, we presented waveguide-integrated plasmonic graphene PDs with 4–5× increased responsivity at cryogenic temperatures, high bandwidth exceeding 45 GHz, and dark current below 200 pA. Next, we combined a 1 × 8 AWG with 8 PDs to demonstrate our cryogenic wavelength-multiplexed optical-to-microwave converter. We characterized all eight device channels and found low electrical crosstalk of less than 30 dB, near-flat frequency response up to the measured 45 GHz, and average channel responsivity of 6 mA/W at 4 K. The low channel responsivity is mainly due to the rather low PD responsivity in the order of 15 mA/W at 4 K or 3 mA/W at RT. Yet, our improved PD design achieved 68 mA/W at RT^45^ and will potentially also benefit from cooldown, bringing the responsivity to a respectable level. Finally, we used our device for a proof-of-concept experiment, where we simultaneously transferred two RF tones modulated onto two different optical carriers into the cryostat. In the best case, we found an isolation between the two channels of >50 dB in electrical power, surpassing current cryogenic multiplexers based on cryo-CMOS achieving 30–40 dB isolation.^20^ This highlights the potential of our approach.

Our device could therefore be a promising solution to enable replacing several bulky RF cables into a cryostat by a single optical fiber. Thereby, this work addresses the much-needed scaling in the number of interconnects from RT to cryogenic environments as required by next-generation quantum computing applications.

## Supplementary Material



## Notes

Literature comparison of cryogenic photodetectors, fabrication process of the devices, modeling of channel shift over temperature in AWG, setup for multiplexed two-tone reception in cryostat (PDF)

## ‡

Zurich Instruments, 8005 Zurich, Switzerland

## §

Schmid & Partner Engineering AG (SPEAG), 8004 Zurich, Switzerland.
