# Supplementary material for: Cryogenic Wavelength-Multiplexed Eight-Channel Optical-to-Microwave Converter
Source: ACS Photonics. 2026 Jul 15;13(15):4317–24. doi: 10.1021/acsphotonics.6c00869 (PMC13450540; doi:10.1021/acsphotonics.6c00869)
Supplement: Supplementary file 1 [file ph6c00869_si_001.pdf]

Supporting Information for

Cryogenic Wavelength-Multiplexed Eight-Channel Optical-to-Microwave Converter

Dominik Bisang*[,1], Stefan M. Koepfli[1], Daniel Rieben[1], Lewin Bormann[1], David Moor[1], Shadi Nashashibi[1], Laurenz Kulmer[1], Michael Baumann[1], Marina Homs[1],Yuriy Fedoryshyn[1], Juerg Leuthold*[,1]

[1]ETH Zurich, Institute of Electromagnetic Fields, 8092 Zurich, Switzerland

*Corresponding Authors: dominik.bisang@ief.ee.ethz.ch, Juerg.Leuthold@ief.ee.ethz.ch

Supporting Note 1: Literature Comparison of Cryogenic Photodetectors

In Table 1, we summarize bandwidth, responsivity, dark current, operation wavelength, measurement temperature, and waveguide-integration of cryogenic high-speed photodetectors we have found in literature.

Table S1. Overview of cryogenic high-speed photodetectors found in the literature.

device and materials	bandwidth (GHz)	responsivity (mA/w)	dark current (pA)	wavelength (nm)	temperature	waveguide-integrated?
GaInAs/InP p-i-n[1]	>20	–	30[a]	1300	9 K	No
commercial InGaAs[2]	–	–	–	1310	4 K	No
commercial InGaAs[3]	10	–	–	1310	4 K	No
modified UTC InGaAs[4]	–	350	<100	1490	20 mK	No
commercial InGaAs[5]	20	500	<10	1490	20 mK	No
metamaterial-graphene[6]	>110	–[b]	0[c]	1560	4 K	No
InGaAs/InP p-i-n[7]	–	500<br>150	<20	1400<br>1550	5 K	Yes
Ge on SOI, type "Vertical"[8]	6	40<br>0.01	140	1308<br>1550	4.2 K	Yes
Ge on SOI, type "Floating"[8]	0.4	150<br>1	0.5	1308<br>1550	4.2 K	Yes
extended-InGaAs[9]	10	850	<10	1550	20 mK	No
commercial silicon[10]	>1	60	<2	904	4.7 K	No
commercial InGaAs[10]	>1	300	<1	1310	4.7 K	No
this work: plasmonic graphene	>45	15	<200	1550	4 K	Yes

[a]Dark current measured at 180 K.

[b]Increase by +12.5 dB in RF power compared to RT is reported, corresponding to a 4.2x increase in responsivity, but no absolute value for the responsivity is stated.

[c]Operated with 0 V bias and 0 V gate voltage.

Supporting Note 2: Fabrication Process of the Devices

The devices presented in this work were fabricated using silicon-on-insulator (SOI) substrate with 725 µm silicon substrate, 3 µm buried oxide (BOX), and 220 nm silicon device layer as starting point. First, e-beam alignment markers were etched into the substrate by using e-beam lithography and dry etching. Next, two silicon etching steps were performed for 70 nm and 220 nm silicon etch, where both steps used e-beam lithography and dry etching. Then, 10 nm Al_2O_3 and 3 µm SiO_2 were deposited as cladding. The chip containing the standalone AWG presented in Figure 2 in the main text was not further processed after this point.

The chip containing the cryogenic eight-channel optical-to-microwave converter, which combines the AWG with the PDs, has undergone the following further processing for co-integration of the PDs: First, the cladding was opened where the PDs should be fabricated by using photolithography, dry etching, and wet etching. Then, a CVD grown bilayer graphene sheet was transferred onto the chip by a wet transfer, followed by patterning of the graphene using e-beam lithography and dry etching. Next, three metal deposition steps were performed by e-beam lithography and lift-off: 30 nm gold for plasmonic resonators; 5 nm titanium and 80 nm gold for probing pads; and 40 nm silver and 40 nm gold for silver contact fingers. The chip was then covered in 150 nm Al_2O_3 for graphene passivation. Next, the top gate consisting of 5 nm titanium and 80 nm gold was deposited by e-beam lithography and lift-off. Finally, the passivation was opened at the probing pads by photolithography and wet etching.

Supporting Note 3: Modelling of Channel Shift over Temperature in AWG

To model how the channel wavelengths in an arrayed waveguide grating (AWG) shift over temperature, we use the equations presented in Ref. [11].

Step 1: Derivation of Equation

We analyze how the central wavelength of the AWG shifts with temperature. The central wavelength λ_c is the wavelength where light propagating through the AWG interferes constructively at the central output. For the central wavelength λ_c in an AWG, the following condition holds (derived from Equation (10) in Ref. [11] with only one type of waveguide, i.e. $\Delta L^{\mathrm{w2}} = 0$):

$$n_{\mathrm{e}}^{\mathrm{b}} \cdot \Delta L^{\mathrm{b}} + n_{\mathrm{e}}^{\mathrm{w}} \cdot \Delta L^{\mathrm{w}} = m\lambda_{\mathrm{c}} \qquad \text{Eq. (1)}$$

Here, $n_{\mathrm{e}}^{\mathrm{b}}$ and $n_{\mathrm{e}}^{\mathrm{w}}$ are the effective mode index of the bends (superscript b) and the straight waveguides (superscript w), ΔL^{b} and ΔL^{w} are the length differences in the bends and straight waveguides between neighboring arms in the AWG, and m is the nominal grating order (a temperature-independent design parameter, $m = 35$ in our design). The bends considered here are the bends directly after the star coupler, which have different length for each arm. The 90°-bends in the "corners" of the arms have equal length in each arm and can be neglected.

The shift of the central wavelength λ_{c} over temperature T is given by (adapted from Equation (13) in Ref. [11]):

$$\frac{\partial \lambda_{\mathrm{c}}}{\partial T} = \frac{1}{M}\left[\frac{\partial n_{\mathrm{e}}^{\mathrm{b}}}{\partial T} \cdot \Delta L^{\mathrm{b}} + \frac{\partial n_{\mathrm{e}}^{\mathrm{w}}}{\partial T} \cdot \Delta L^{\mathrm{w}}\right] \qquad \text{Eq. (2)}$$

with M being the modified grating order defined as (adapted from Equation (11) in Ref. [11]):

$$M = m \cdot \frac{n_{\mathrm{g}}^{\mathrm{b}} \cdot \Delta L^{\mathrm{b}} + n_{\mathrm{g}}^{\mathrm{w}} \cdot \Delta L^{\mathrm{w}}}{n_{\mathrm{e}}^{\mathrm{b}} \cdot \Delta L^{\mathrm{b}} + n_{\mathrm{e}}^{\mathrm{w}} \cdot \Delta L^{\mathrm{w}}} \qquad \text{Eq. (3)}$$

Here, $n_{\mathrm{g}}^{\mathrm{b}}$ and $n_{\mathrm{g}}^{\mathrm{w}}$ are the group index of the modes in the bends and straight waveguides.

We now fix all values at the reference temperature T_0 and consider a shift in temperature $\Delta T = T - T_0$. According to Eq. (2), the central wavelength shifts by $\Delta\lambda_{\mathrm{c}}$:

$$\Delta\lambda_{\mathrm{c}} = \lambda_{\mathrm{c}}(T) - \lambda_{\mathrm{c}}(T_0) = \frac{1}{M}\left[\Delta n_{\mathrm{e}}^{\mathrm{b}} \cdot \Delta L^{\mathrm{b}} + \Delta n_{\mathrm{e}}^{\mathrm{w}} \cdot \Delta L^{\mathrm{w}}\right] \qquad \text{Eq. (4)}$$

with $\Delta n_{\mathrm{e}}^{\mathrm{b}} = n_{\mathrm{e}}^{\mathrm{b}}(T) - n_{\mathrm{e}}^{\mathrm{b}}(T_0)$ and $\Delta n_{\mathrm{e}}^{\mathrm{w}} = n_{\mathrm{e}}^{\mathrm{w}}(T) - n_{\mathrm{e}}^{\mathrm{w}}(T_0)$, respectively. Please note that M is also a function of temperature, since $n_{\mathrm{g}}^{\mathrm{b}}$, $n_{\mathrm{g}}^{\mathrm{w}}$, $n_{\mathrm{e}}^{\mathrm{b}}$ and $n_{\mathrm{e}}^{\mathrm{w}}$ depend on the temperature. Additionally, ΔL^{b} and ΔL^{w} also slightly change with temperature. However, this change is significantly lower than the change in the effective mode index and group index, which is why it has been neglected in the derivation above. This will be reintroduced later by making ΔL^{b} and ΔL^{w} a function of temperature, see Step 3 further below.

Step 2: Effective Mode Index and Group Index over Temperature

To find $n_{\mathrm{g}}^{\mathrm{b}}$, $n_{\mathrm{g}}^{\mathrm{w}}$, $n_{\mathrm{e}}^{\mathrm{b}}$ and $n_{\mathrm{e}}^{\mathrm{w}}$ as a function of temperature, we perform 2D mode simulations in Ansys Lumerical MODE. For this, we first need the refractive index of the materials as a function of temperature and wavelength. We implement the materials for silicon and silicon dioxide using Lumerical's built-in wavelength-dependent materials based on data provided by Palik.[12] The temperature dependence is then added using index perturbation, where we use data from Ref. [13] for silicon and from Ref. [14] for silicon dioxide. The resulting refractive index for silicon and silicon dioxide at 1550 nm wavelength are shown in Figure S1.

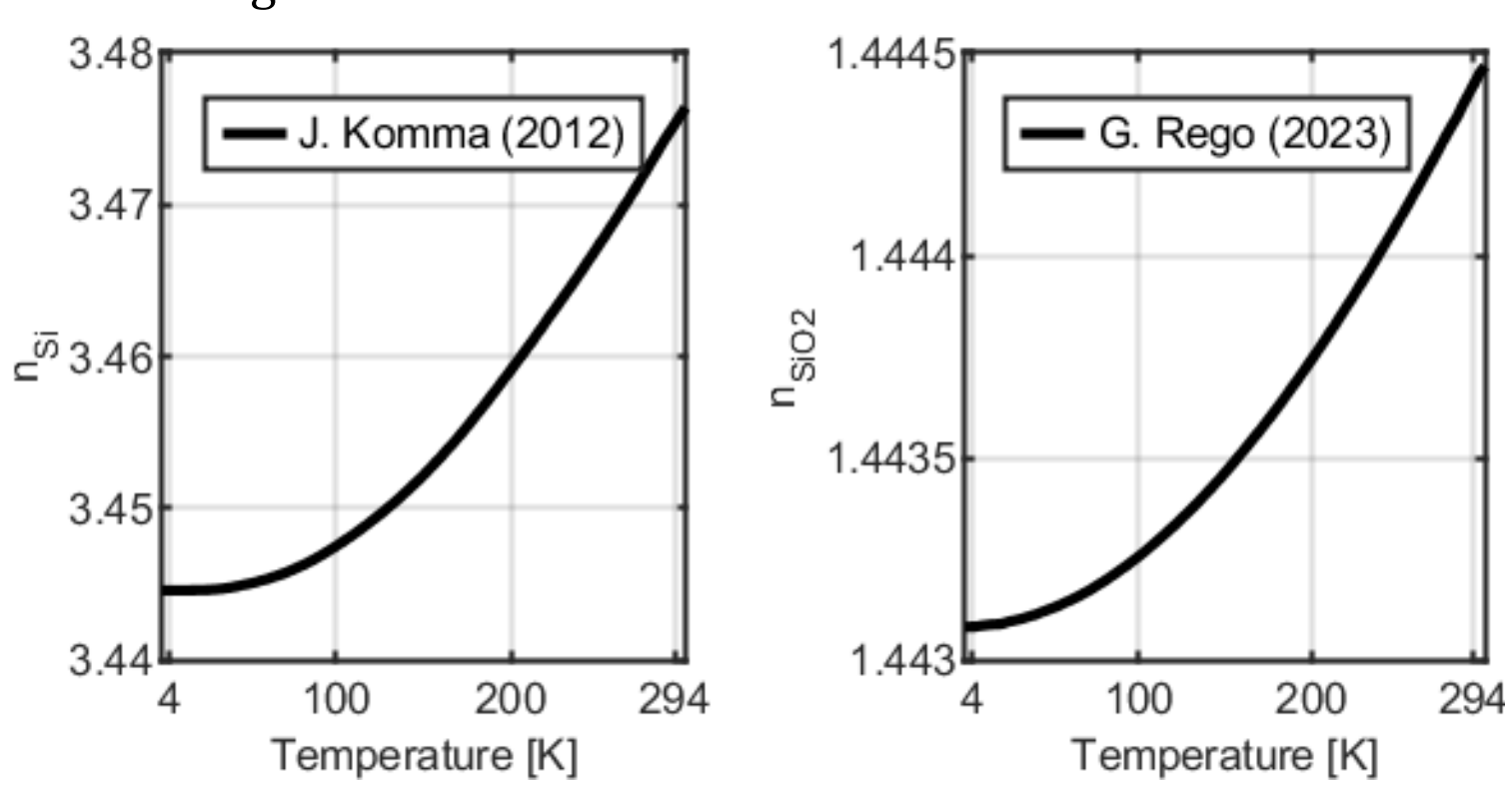

Figure S1. Refractive index of (left) silicon and (right) silicon dioxide at 1550 nm as a function of temperature. Data for silicon taken from Ref. [13] and for silicon dioxide from Ref. [14].

For a thin 10 nm layer of Al_2O_3 which is present on the chip with the standalone AWG (Figure 2(a) in the main text), we assumed a constant index of 1. 6349 for all wavelengths and temperatures. This assumption is made because we have found conflicting values for the refractive index of Al_2O_3 thin films in literature, therefore we took an average over five different sources from literature. Further, we have not found temperature dependence for Al_2O_3 thin films in literature. More precise material values might improve our model, but for a 10 nm thin layer we assume the temperature-induced changes to be negligible.

Having all the temperature-dependent materials available, we perform 2D mode simulations in Ansys Lumerical MODE. The waveguide geometry used for the simulation is shown in Figure S2. The parameters used are: $w_{\mathrm{WG}} = w_{\mathrm{Rib}} = 700\ \mathrm{nm}$, $h_{\mathrm{WG}} = 220\ \mathrm{nm}$, $h_{\mathrm{Rib}} = 150\ \mathrm{nm}$, $h_{\mathrm{Al2O3}} = 10\ \mathrm{nm}$, $h_{\mathrm{BOX}} = 3\ \mathrm{\mu m}$. The bend radius of the strip bends in Figure S2(a) is 20 µm. The resulting simulated effective mode index and group index of bends and straight waveguides are shown in Figure S3.

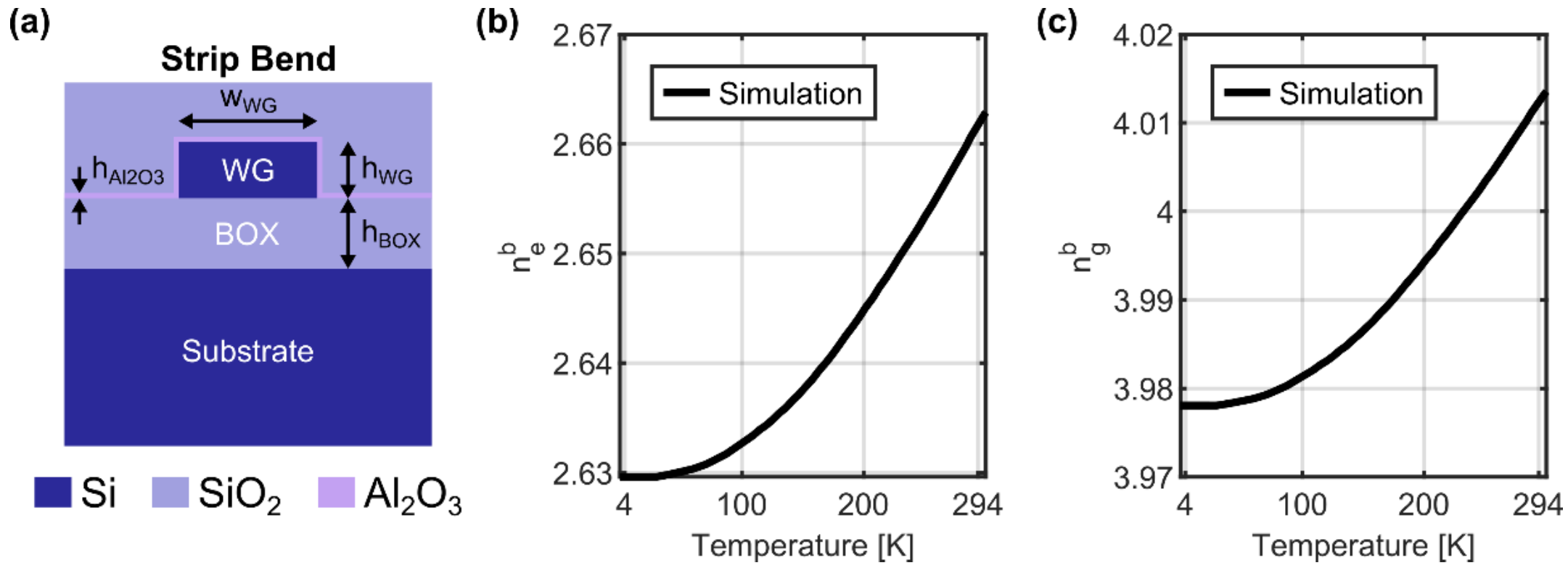

Figure S2. Strip waveguide in the bends after the star coupler of the AWG. (a) Schematic cross-section used for the simulation. (b) Simulated effective mode index $n_{\mathrm{e}}^{\mathrm{b}}$ over temperature. (c) Simulated group index $n_{\mathrm{g}}^{\mathrm{b}}$ over temperature.

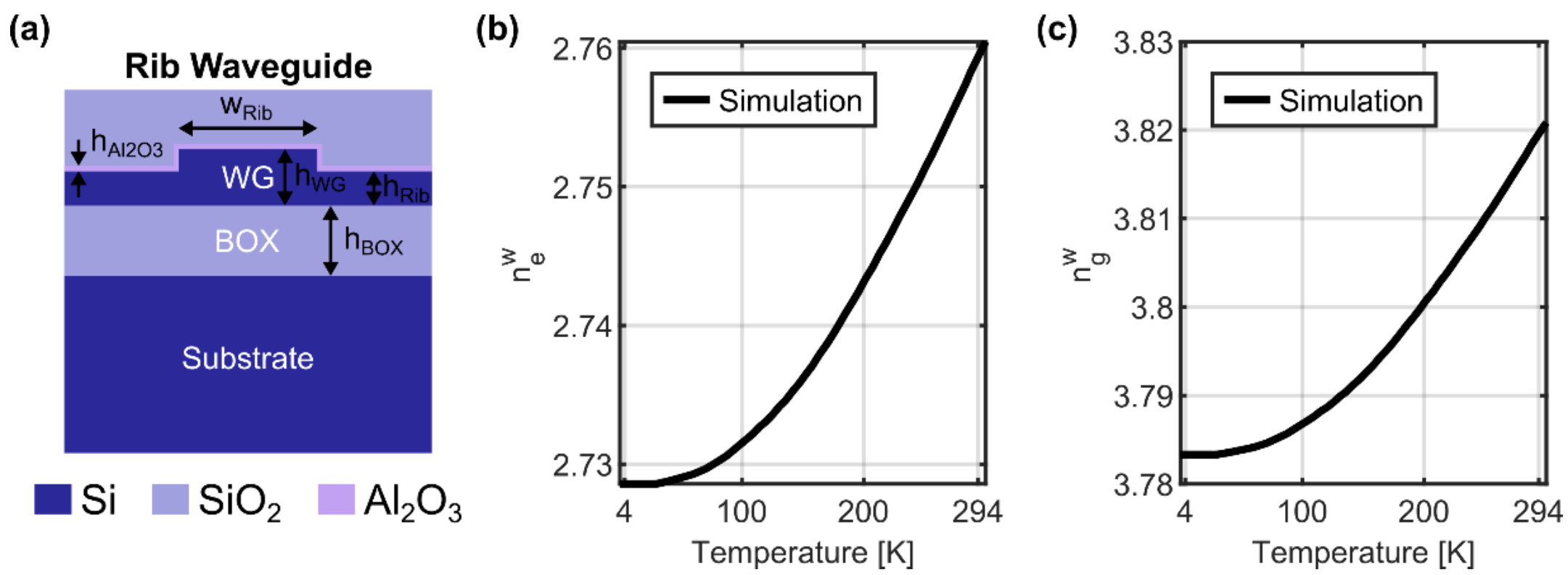

Figure S3. Rib waveguides in the straight sections of the AWG. (a) Schematic cross-section used for the simulation. (b) Simulated effective mode index $n_{\mathrm{e}}^{\mathrm{w}}$ over temperature. (c) Simulated group index $n_{\mathrm{g}}^{\mathrm{w}}$ over temperature.

Step 3: Length Contraction over Temperature

Now we consider the length contraction due to temperature. This will lead to a temperature-dependence of ΔL^{b} and ΔL^{w}. Since both the chip substrate and the waveguides are made of silicon, we consider the thermal expansion of silicon as the determining factor for the length contraction. The linear thermal expansion coefficient of silicon α_{Si} with units 1/K is given by Ref. [15]. Integrating over temperature, we get the relative length of silicon compared to 0 K or $\eta_{\mathrm{Si,0K}}$:

$$\eta_{\mathrm{Si,0K}}(T) = 1 + \int_{0\ \mathrm{K}}^{T} \alpha_{\mathrm{Si}}(T')\, \mathrm{d}T' \qquad \text{Eq. (5)}$$

Since we know the designed and fabricated lengths at room temperature, we consider the relative length of silicon compared to 294 K or $\eta_{\mathrm{Si,294K}}$:

$$\eta_{\mathrm{Si,294K}}(T) = \frac{\eta_{\mathrm{Si,0K}}(T)}{\eta_{\mathrm{Si,0K}}(294\ \mathrm{K})} \qquad \text{Eq. (6)}$$

Figure S4 visualizes α_{Si}, $\eta_{\mathrm{Si,0K}}(T)$ and $\eta_{\mathrm{Si,294K}}(T)$ over temperature.

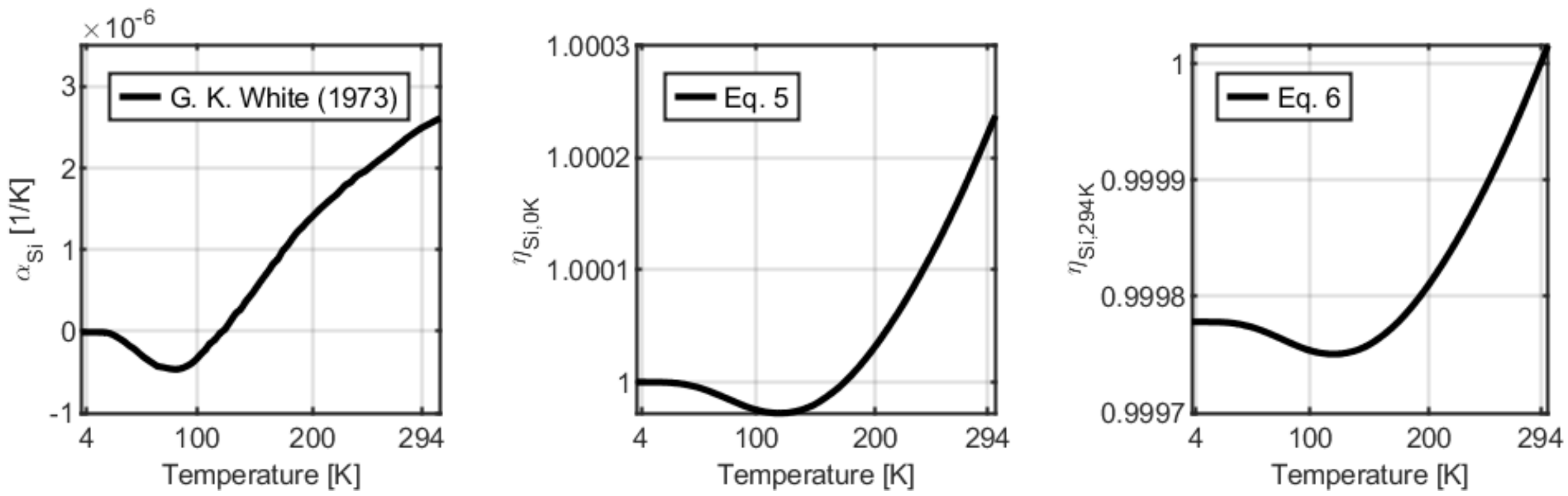

Figure S4. (Left) Linear thermal expansion coefficient α_{Si} of silicon as given by Ref. [15]. (Center, Right) Relative length of silicon compared to the length at (center) 0 K and (right) 294 K.

Finally, we can express ΔL^b and ΔL^w as functions of temperature:

$$\Delta L^b(T) = \eta_{\mathrm{Si,294K}}(T) \cdot \Delta L^b(294\ \mathrm{K}) \quad \text{with} \quad \Delta L^b(294\ \mathrm{K}) = -0.425\ \mathrm{\mu m} \qquad \text{Eq. (7)}$$

$$\Delta L^w(T) = \eta_{\mathrm{Si,294K}}(T) \cdot \Delta L^w(294\ \mathrm{K}) \quad \text{with} \quad \Delta L^w(294\ \mathrm{K}) = 20.876\ \mathrm{\mu m} \qquad \text{Eq. (8)}$$

However, the temperature-induced change in length is significantly smaller than the change in the effective mode index and group index, which is why the length contraction could also be neglected here.

Step 4: Resulting Model of AWG Channel Shift over Temperature

Using Eq. (4) together with the simulated effective mode index and group index from Figure S2, and with the relative length change from Eq. (7) and Eq. (8), we can model the AWG channel shift over temperature. The modelled channel shift is shown in Figure S5, which is the same data as Figure 2(c) in the main text.

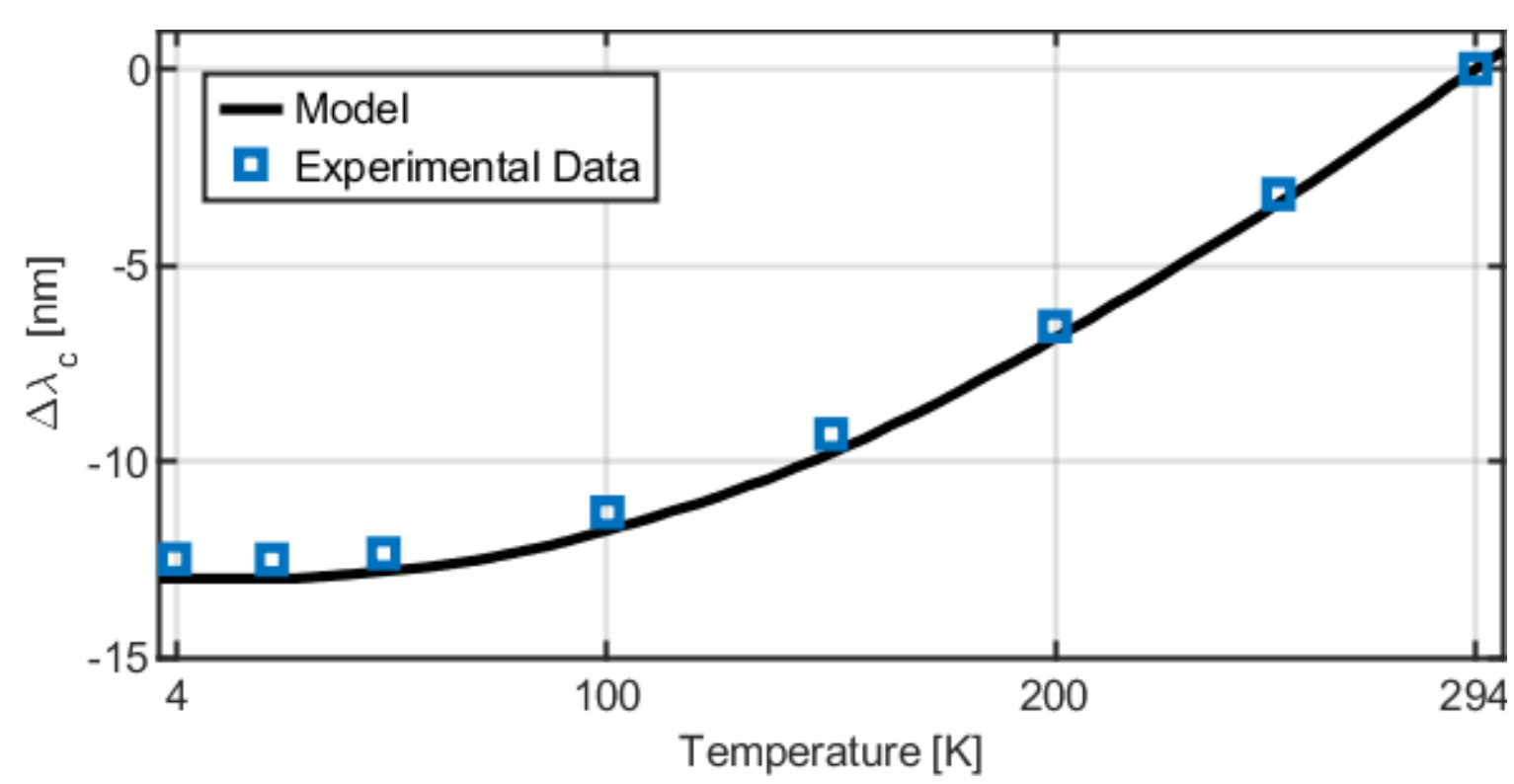

Figure S5. Model (black solid line) and experimental data (blue squares) of channel shift of the AWG $\Delta\lambda_c$ vs temperature.

Towards low temperatures, a small deviation of around 4% in the model is present (-12.5 nm shift measured, -13.0 nm shift modelled). The main reason in this deviation is most likely uncertainty in the parameters: First, deviations in the fabricated geometry from the designed geometry will impact the effective mode index and group index. This can be improved by a well-controlled fabrication environment and extensive metrology of the fabricated devices. Second, the material parameters have some uncertainties. The refractive index of SiO_2 used in the model is for bulk material and can deviate for thin films. Also, the refractive index of Al_2O_3 is only an estimate. By performing ellipsometry on deposited thin films, these values can be determined more precisely. Additionally, there is also uncertainty in the experimental data such as the exact temperature of the device or the extracted peak wavelength of the AWG channel.

Nevertheless, the model and the experimental data are in good agreement over a large temperature range with only a small deviation. This indicates that the theory of operation for AWGs is well understood and can be extended to cryogenic temperatures.

Supporting Note 4: Discussion on Active Heat Load

Here, we briefly discuss the active heat load imposed by our eight-channel optical-to-microwave converter to the cryogenic environment. A typical cryostat suited for superconducting qubits has only around 1 W of cooling power at the 4 K stage,[16] where our converter would be operating. For scaling to as many channels as possible, it is therefore important to keep the active heat load by the optical-to-microwave conversion low. In the subsequent discussion, we neglect passive heat load from electrical cabling and optical fibers, since these are independent of our specific optical-to-microwave converter and are expected to be low by using photonic links.

The active heat load depends on the required signal amplitude reaching the qubit, which is typically -80 to -60 dBm (0.01 to 1 µW) for superconducting qubits[17]. For a single channel in our current experiments, we need an optical power in the fiber entering the cryostat $P_{\text{opt,In}}$ of around +16 dBm (40 mW) for the generation of an electrical signal with RF power P_{RF} of -62 dBm (0.63 µW) at the output of the PD. Therefore, most of the incoming power is lost, giving currently an active heat load of ≈40 mW per channel, or ≈320 mW if all eight channels are operated simultaneously. If another RF power level than -62 dBm is required or additional attenuators are present between the qubits and the optical-to-microwave converter, then the optical input power and resulting active heat loads need to be scaled accordingly.

However, the active heat load can be massively improved. Currently, three loss mechanisms dominate the active heat load in our optical-to-microwave converter: Inefficient fiber-to-chip coupling T_{Coupling} of about 0.1 (-10 dB), AWG transmission T_{AWG} of 0.6 (-2.2 dB) on average, and low PD responsivity $\mathcal{R}_{\text{PD}}$ of typical around 10 mA/W. The AWG transmission and PD responsivity together form the channel responsivity $\mathcal{R}_{\text{Channel}} = T_{\text{AWG}} \cdot \mathcal{R}_{\text{PD}}$ of 6 mA/W on average, which is reported in Figure 4(d) in the main text. These three loss mechanisms are listed in column (A) of Table S2.

The RF power P_{RF} at the PD output scales with the photocurrent I_{ph} squared, i.e., $P_{\text{RF}} \sim I_{\text{ph}}^2$. Since the photocurrent is optical power in the channel $P_{\text{opt,Channel}}$ multiplied with channel responsivity $\mathcal{R}_{\text{Channel}}$; and the optical power in the channel is given by $P_{\text{opt,Channel}} = T_{\text{Coupling}} \cdot P_{\text{opt,In}}$; we find for the RF power:

$$P_{\text{RF}} \sim \left(\mathcal{R}_{\text{PD}} \cdot T_{\text{AWG}} \cdot T_{\text{Coupling}} \cdot P_{\text{opt,In}}\right)^2 \qquad \text{Eq. (9)}$$

Therefore, every 1 dB improvement in T_{Coupling}, T_{AWG}, or $\mathcal{R}_{\text{Channel}}$ will allow that $P_{\text{opt,In}}$ can be reduced by 2 dB to achieve the same RF power at the PD output.

Table S2. Loss mechanisms determining the active head load.

parameter	(A) typical values in experiment	(B) optimized design	(C) best values from literature
T_{Coupling} fiber-to-chip coupling efficiency	0.1 (-10 dB)	0.5 (-3 dB)	0.9881 (-0.052 dB)[18]
T_{AWG} AWG transmission	0.6 (-2.2 dB)	0.8 (-1 dB)	0.885 (-0.53 dB)[19]
$\mathcal{R}_{\text{PD}}$ PD responsivity	10 mA/W	200 mA/W	850 mA/W[9]
**$P_{\text{opt,In}}$ required optical power entering cryostat for -62 dBm RF power after PD**	**40 mW (+16 dBm)**	**2.3 µW (-26 dBm)**	**21 nW (-47 dBm)**
**active heat load of 8-channel optical-to-microwave converter**	**320 mW**	**18.4 µW**	**168 nW**

Let us now consider a scenario with improved designs of our components. By employing our improved graphene PD design showing 68 mA/W in C-band and 200 mA/W in L-band at room temperature[20] and assuming that its responsivity also increases during cooldown, we assume a cautiously optimistic PD responsivity of 0.200 mA/W. Additionally, we assume that the fiber-to-chip coupling losses could be reduced to -3 dB and AWG transmission loss reduced to -1 dB. In that case, according to Eq. (9), the required optical input power could be reduced by 42 dB down to -26 dBm to achieve the same RF power. This would result in an active heat load of 18.4 µW for a -62 dBm RF signal at the output of all eight channels. These values are summarized in column (B) of Table S2.

When considering the best reported values in literature, even lower active heat loads could be achieved. Fiber-to-chip coupling as high as 0.9881 (-0.052 dB) has been demonstrated for silicon photonics.[18] For AWGs in silicon photonics, insertion loss of -0.53 dB have been reported in the C-band.[19] Regarding PDs, the highest responsivity reported in C-band at cryogenic temperatures is 850 mA/W.[9] We note that this particular PD is not waveguide-integrated, as would be required for dense co-integration with WDM, but we are optimistic that these responsivity values can be achieved

in waveguide-integrated PDs in silicon photonics. Using these best values, summarized in column (C) of Table S2, the optical input power could be reduced by 63 dB down to -47 dBm, resulting in 168 nW active heat load by the optical-to-microwave converter. At that point, other heat sources such as RF loss or passive heat load from cables will most likely play a role as well.

Finally, we want to note that in quantum computing applications, each channel would not be active all the time, but only while sending control signals to the qubits. This average usage time is denoted as duty cycle or activity rate. If the duty cycle is sufficiently low, the active heat load can be reduce by more than a factor 1000 in principle.[5] Yet, more realistic approaches assume duty cycles of around 33% per channel,[16,21,22] thereby reducing the previously stated values by a factor of 3.

Overall, this analysis highlights the potential of our approach. By reducing especially fiber-to-chip coupling loss and improving PD responsivity by using our improved graphene PD design, the active heat load can be massively reduced.

Supporting Note 5: Setup for Multiplexed Two-Tone Reception in Cryostat

Figure S5 shows a detailed schematic of the setup used for the multiplexed two-tone reception in the cryostat (Figure 5 in the main text). To combine the two modulated signals, a 50:50 splitter was used, where one output was fed to the subsequent amplifier and the other output was left unconnected. Care was taken to bring the two signals after the combiner to the same power levels and same polarization, to compensate for differences between the two modulators and give a better comparison of the two DUT channels.

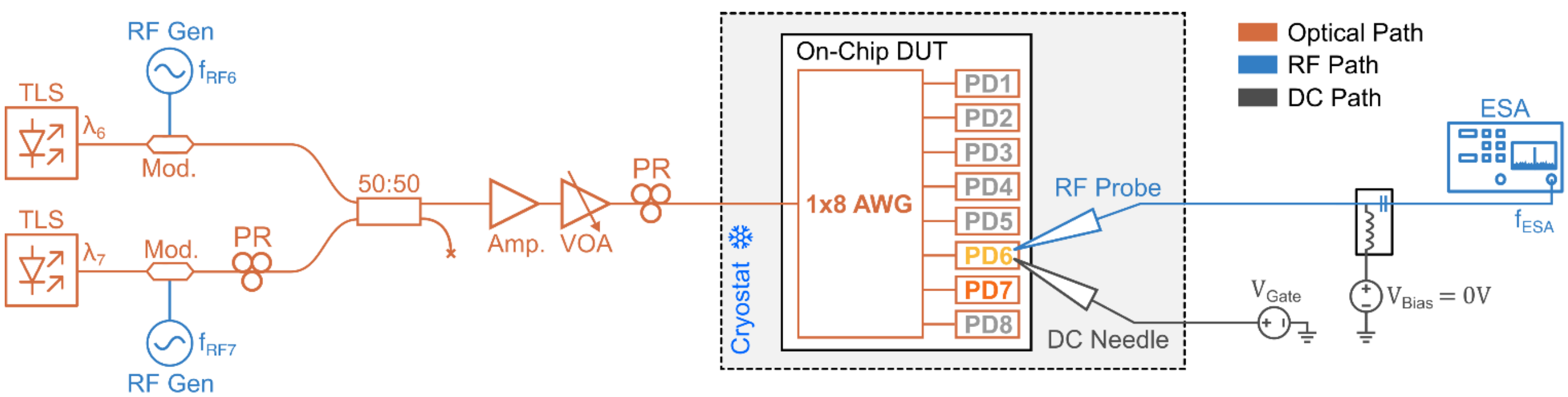

Figure S5. Detailed schematic of the setup used for the multiplexed two-tone reception experiment inside the cryostat. TLS, tunable laser source; RF Gen, RF generator; Mod., modulator; PR, polarization rotator; Amp., amplifier; VOA, variable optical attenuator; DUT, device under test; AWG, arrayed waveguide grating; PD, photodetector; ESA, electrical spectrum analyzer.

References

(1) Zhang, Y. M.; Borzenets, V.; Dubash, N.; Reynolds, T.; Wey, Y. G.; Bowers, J. Cryogenic Performance of a High-Speed GaInAs / InP p-i-n Photodiode. *Journal of Lightwave Technology* **1997**, *15* (3), 529–533. https://doi.org/https://doi.org/10.1109/50.557569.

(2) Gran, J.; Malmbekk, H.; Lind, K. Photodiodes as Current Source in High-Frequency Low Temperature Applications. *CPEM Digest (Conference on Precision Electromagnetic Measurements)* **2014**, 266–267. https://doi.org/10.1109/CPEM.2014.6898361.

(3) Bardalen, E.; Karlsen, B.; Malmbekk, H.; Akram, M. N.; Ohlckers, P. Evaluation of InGaAs/InP Photodiode for High-Speed Operation at 4 K. *Int. J. Metrol. Qual. Eng.* **2018**, *9*, 13. https://doi.org/10.1051/ijmqe/2018015.

(4) Davila-Rodriguez, J.; Teufel, J. D.; Aumentado, J. A.; Xie, X.; Campbell, J. C.; Diddams, S. A.; Quinlan, F. High-Speed Photodetection and Microwave Generation in a Sub-100 mK Environment. In *Conference on Lasers and Electro-Optics*; OSA: San Jose, California, 2019; p SF2N.1. https://doi.org/10.1364/CLEO_SI.2019.SF2N.1.

(5) Lecocq, F.; Quinlan, F.; Cicak, K.; Aumentado, J.; Diddams, S. A.; Teufel, J. D. Control and Readout of a Superconducting Qubit Using a Photonic Link. *Nature* **2021**, *591* (7851), 575–579. https://doi.org/10.1038/s41586-021-03268-x.

(6) Koepfli, S. M.; Bisang, D.; Kulmer, L.; Baumann, M.; Nashashibi, S.; Rieben, D.; Peter, P.; Horst, Y.; Fedoryshyn, Y.; Leuthold, J. Ambient to Cryogenic High-Frequency Response of Zero-Bias Graphene Photodetectors. *ACS Appl. Mater. Interfaces* **2026**. https://doi.org/10.1021/acsami.6c05931.

(7) Iadanza, S.; Rihani, M.; Martinez, C. O.; Scherrer, M. A.; Georgiev, V.; Schmid, H.; Moselund, K. E. Ultra-Low Temperature Characterization of Fully-Integrated III-V Photodetectors for Quantum Networks. *2024 IEEE International Electron Devices Meeting (IEDM)* **2024**, 1–4. https://doi.org/10.1109/IEDM50854.2024.10873440.

(8) Julien-Neitzert, D.; Leung, E. K.; Islam, N.; Khorev, S.; Shekhar, S.; Chrostowski, L.; Young, J. F.; Salfi, J. Cryogenic Optical-to-Microwave Conversion Using Si Photonic Integrated Circuit Ge Photodiodes. *APL Photonics* **2024**, *9* (11), 116107. https://doi.org/10.1063/5.0223360.

(9) Nakamura, T.; Lee, D.; Horng, J.; Lecocq, F.; Teufel, J.; Quinlan, F. Cryogenic Photonic Link Using an Extended-InGaAs Photodiode and Short Pulse Illumination toward High-Fidelity Drive of Superconducting Qubits. *Optica Quantum, OPTICAQ* **2025**, *3* (3), 221–227. https://doi.org/10.1364/OPTICAQ.546795.

(10) Mutum, S.; Vliex, P.; Bühler, J.; Nielinger, D.; Schlösser, M.; van Waasen, S. A Photonic Link at 4.7 K with >1 GHz Bandwidth Towards an Optical Quantum Computing Interface. In *2025 IEEE/MTT-S International Microwave Symposium - IMS 2025*; 2025; pp 73–76. https://doi.org/10.1109/IMS40360.2025.11103848.

(11) Huang, H.; Ho, S.-T.; Huang, D.; Tu, Y.; Liu, W. Design of Temperature-Independent Arrayed Waveguide Gratings Based on the Combination of Multiple Types of Waveguide. *Appl. Opt., AO* **2010**, *49* (16), 3025–3034. https://doi.org/10.1364/AO.49.003025.

(12) Palik, E. D. *Handbook of Optical Constants of Solids*; Academic Press, 1985; Vol. Volume 1.

(13) Komma, J.; Schwarz, C.; Hofmann, G.; Heinert, D.; Nawrodt, R. Thermo-Optic Coefficient of Silicon at 1550 Nm and Cryogenic Temperatures. *Applied Physics Letters* **2012**, *101* (4), 041905. https://doi.org/10.1063/1.4738989.

(14) Rego, G. Temperature Dependence of the Thermo-Optic Coefficient of SiO2 Glass. *Sensors* **2023**, *23* (13), 6023. https://doi.org/10.3390/s23136023.

(15) White, G. K. Thermal Expansion of Reference Materials: Copper, Silica and Silicon. *J. Phys. D: Appl. Phys.* **1973**, *6* (17), 2070. https://doi.org/10.1088/0022-3727/6/17/313.

(16) Krinner, S.; Storz, S.; Kurpiers, P.; Magnard, P.; Heinsoo, J.; Keller, R.; Lütolf, J.; Eichler, C.; Wallraff, A. Engineering Cryogenic Setups for 100-Qubit Scale Superconducting Circuit Systems. *EPJ Quantum Technology* **2019**, *6*. https://doi.org/10.1140/EPJQT/S40507-019-0072-0.

(17) Bardin, J. C.; Slichter, D. H.; Reilly, D. J. Microwaves in Quantum Computing. *IEEE Journal of Microwaves* **2021**, *1* (1), 403–427. https://doi.org/10.1109/JMW.2020.3034071.

(18) Alexander, K.; Benyamini, A.; Black, D.; Bonneau, D.; Burgos, S.; Burridge, B.; Cable, H.; Campbell, G.; Catalano, G.; Ceballos, A.; Chang, C.-M.; Choudhury, S. S.; Chung, C. J.; Danesh, F.; Dauer, T.; Davis, M.; Dudley, E.; Er-Xuan, P.; Fargas, J.; Farsi, A.; Fenrich, C.; Frazer, J.; Fukami, M.; Ganesan, Y.; Gibson, G.; Gimeno-Segovia, M.; Goeldi, S.; Goley, P.; Haislmaier, R.; Halimi, S.; Hansen, P.; Hardy, S.; Horng, J.; House, M.; Hu, H.; Jadidi, M.; Jain, V.; Johansson, H.; Jones, T.; Kamineni, V.; Kelez, N.; Koustuban, R.; Kovall, G.; Krogen, P.; Kumar, N.; Liang, Y.; LiCausi, N.; Llewellyn, D.; Lokovic, K.; Lovelady, M.; Manfrinato, V. R.; Melnichuk, A.; Mendoza, G.; Moores, B.; Mukherjee, S.; Munns, J.; Musalem, F.-X.; Najafi, F.; O'Brien, J. L.; Ortmann, J. E.; Pai, S.; Park, B.; Peng, H.-T.; Penthorn, N.; Peterson, B.; Peterson, G.; Poush, M.; Pryde, G. J.; Ramprasad, T.; Ray, G.; Rodriguez, A. V.; Roxworthy, B.; Rudolph, T.; Saunders, D. J.; Shadbolt, P.; Shah, D.; Bahgat Shehata, A.; Shin, H.; Sinsky, J.; Smith, J.; Sohn, B.; Sohn, Y.-I.; Son, G.; Souza, M. C. M. M.; Sparrow, C.; Staffaroni, M.; Stavrakas, C.; Sukumaran, V.; Tamborini, D.; Thompson, M. G.; Tran, K.; Triplett, M.; Tung, M.; Veitia, A.; Vert, A.; Vidrighin, M. D.; Vorobeichik, I.; Weigel, P.; Wingert, M.; Wooding, J.; Zhou, X.; PsiQuantum team. A Manufacturable Platform for Photonic Quantum Computing. *Nature* **2025**, *641* (8064), 876–883. https://doi.org/10.1038/s41586-025-08820-7.

(19) Pathak, S.; Thourhout, D. V.; Bogaerts, W. Design Trade-Offs for Silicon-on-Insulator-Based AWGs for (de)Multiplexer Applications. *Opt. Lett., OL* **2013**, *38* (16), 2961–2964. https://doi.org/10.1364/OL.38.002961.

(20) Rieben, D.; Blatter, T.; Koepfli, S. M.; Kulmer, L.; Horst, Y.; Moor, D.; Homs, M.; Bisang, D.; Baumann, M.; Fedoryshyn, Y.; Leuthold, J. Towards 200 GBaud Line Rates with Waveguide-Integrated Plasmonic Graphene Photodetectors. In *Optical Fiber Communication Conference (2025), paper Th3E.4*; 2025.

(21) Joshi, S.; Moazeni, S. Scaling up Superconducting Quantum Computers With Cryogenic RF-Photonics. *Journal of Lightwave Technology* **2024**, *42* (1), 166–175. https://doi.org/10.1109/JLT.2023.3311806.

(22) Succar, M.; Ibrahim, M. I. Ingress Cryogenic Receivers Toward Scalable Quantum Information Processing: Theory and System Analysis. arXiv September 30, 2025. https://doi.org/10.48550/arXiv.2509.25768.
